# Innovameter: Agent-based modeling of innovation determinants in American and European countries

**DOI:** 10.1371/journal.pone.0313756

**Published:** 2025-01-07

**Authors:** Arles Rodríguez, Mercedes Gaitán-Angulo, Melva Inés Gómez-Caicedo, Paula Robayo-Acuña, Iván Ricardo Ruíz-Castro

**Affiliations:** 1 Departamento de Matemáticas, Facultad de Ciencias, Universidad Nacional de Colombia, Bogotá, Colombia; 2 Escuela de Negocios, Fundación Universitaria Konrad Lorenz, Bogotá, Colombia; 3 Escuela de Posgrados, Fundación Universitaria Konrad Lorenz, Bogotá, Colombia; 4 Facultad de Economía y Ciencias Contables, Fundación Universitaria Los Libertadores, Bogotá, Colombia; Fooyin University, TAIWAN

## Abstract

This article discusses the dynamics of innovation in America and Europe, focusing on variables such as access to technology, education, and life expectancy. To do this, the article proposes an agent-based model called the Innovameter. The dependent variable is the Global Innovation Index. The paper focuses on data analysis through correlation analysis and multiple hierarchical regressions to determine the contribution of specific variables related to the pillars of the Global Innovation Index and indicators of the Human Development Index. After analyzing the data, an agent-based model was built to parameterize these main variables by defining two levels of abstraction: at the global level, there is the country, where birth rates, life expectancy, ICT use, and research and development are defined. At the local level, we define the individuals who have an age, years of schooling, and income. A series of experiments were conducted by selecting data from 30 countries. From the results of the experiments, a nonparametric correlation analysis was performed, and correlation indices were obtained indicating a relationship between the predicted outcomes and the outcomes in the global index. The proposed model aims to provide suggestions on how the different variables can become the norm in most of the countries studied.

## Introduction

Innovation can be divided into two dimensions: The first is focused on production and mass consumption, where its characteristics focus on making organizations more productive and where government plays a key role. The second dimension is based on economic growth, where competitiveness, internationalization of markets and globalization are identified as key factors, and challenges such as eradicating poverty and reducing inequality in all its forms in the world are generated, for which the generation of a production and consumption system that is inclusive, sustainable and able to face climate change is proposed, not only achieving the satisfaction of social needs but also creating the need to take care of the planet for future generations [1].

However, according to the WIPO [2] Global Innovation Index report, the COVID-19 pandemic has brought with it a slowdown in productivity growth and other ever-changing challenges. In fact, this slowdown in indicators of the incidence of innovation, such as productivity growth—the parameter used by economists to assess whether living standards are likely to improve over time—is at its lowest level in history. The so-called period of great stagnation calls into question the ability of innovation to generate growth in the future.

On the other hand, other barriers to technology adoption and diffusion need to be overcome and countries need to develop new innovation strategies and economic policies to improve productivity, economic growth, and social welfare.

Only a few countries perform better in innovation, including the top three innovative economies in Europe: Switzerland, Sweden, and the United Kingdom; the United States and Canada in North America; and Chile, Brazil and Mexico in Latin America and the Caribbean. South Africa, Botswana and Kenya also stand out in sub-Saharan Africa, while Israel, the United Arab Emirates and Turkey stand out in North Africa and East Asia. In Southeast Asia—East Asia are the Republic of Korea, Singapore, and China, and finally in Central and South Asia with India, the Islamic Republic of Iran and Uzbekistan as leaders for this region.

As for the overall global assessment, the list of the top 15 countries leading in innovation are Switzerland, United States of America, Sweden, United Kingdom, Netherlands, Republic of Korea, Singapore, Germany, Finland, Denmark, China, France, Japan, Hong Kong, and Canada. These countries show those global trends in the context and the moment in which the world is, in terms of the situation experienced in the year 2020 to 2022 with the pandemic of COVID-19, which provided the opportunity to innovate in a constant and accelerated way to meet the needs of people.

Despite advances in understanding the factors that influence innovation at the country [3, 4], there is a notable gap in the extant literature in terms of models that can dynamically and holistically simulate the complex interaction of multiple variables that contribute to a country’s innovative capacity. Extant literature, as evidenced by studies such as those conducted by Ulku [5], Crespi & Zuñiga [6] and Lootty [7], indicates that the determinants of innovation are not uniform across all countries. Consequently, disparities in innovation patterns have been identified between developed and developing countries. Additionally, the significance of territorial innovation for economic growth and quality of life has been underscored by numerous studies. Conversely, the extant models for measuring innovation are subject to limitations, frequently exhibiting a lack of dynamism that is requisite for capturing the evolving nature of innovation processes. An integrated approach that can model the interaction of factors such as education, income, life expectancy, technology use, and R&D investment at the individual and national levels is currently lacking.

Embuz and Fernandez-Ledesma [8] posit that agent-based simulation models, which have been widely utilized in other fields, may offer novel insights into the study of innovation. Moreover, Bate et al. [3] highlight the necessity for interdisciplinary approaches that integrate economic, social, and technological perspectives. Current indices, such as the Global Innovation Index, provide a static representation of innovation outcomes, lacking the capacity to predict the impact of changes in these variables on long-term innovation outcomes. The lack of adaptable models that align with diverse national or regional contexts impedes the development of effective and tailored innovation policies.

González-Bailón [9], Embuz & Fernández-Ledesma [8] show that agent-based modeling allows generating advantages for the study of social interactions, in this type of research there is a very marked absence of complex empirical data on interaction networks between agents, where millions of nodes can arise, simulation can be implemented to study the impact of the structure of such networks on all the dynamic processes that take place within it. On the other hand, to date, business science research has only focused on data science and business problem analysis [10], and this is not an exception for innovation research, hence there are not many studies on innovation using agent-based modeling. Other studies have applied the agent-based modeling technique as a way to understand the dynamics of a complex system and simulate its evolution over time [11], but not many of them have focused on the factors that directly affect innovation in countries.

Mainly, the works developed from the ABM in the field of innovation have started from the work of Rogers [12, 13], who studied the diffusion behavior of innovations in individuals, and which has allowed to derive other works related to this particular topic, such as the one presented by Desmarchelier & Fang [14], who carried out a theoretical research on the role of national culture in shaping the diffusion patterns of innovations in different markets, based on the construction of an agent-based cultural model from two of Hofstede’s cultural dimensions (individualism/collectivism and uncertainty avoidance). Similarly, the study by He & Lee [15], in which they applied an agent-based model, focused on investigating the effect of social culture on the diffusion of innovation by incorporating cultural factors in the social structure and individual characteristics.

Another study that differs from the diffusion topic is the one developed by Lopolito et al. [16], in which they moderated the emergence of an innovation niche based on three niche mechanisms: expectations, networking and learning. And they evaluated the impact of an information campaign and the granting of subsidies, in order to confirm the importance of political intervention to guide the process of creation of these innovation niches.

The book "Innovation Networks for Regional Development. Concepts, Case Studies, and Agent-Based Models" [17] takes a multidisciplinary perspective on regional development and the role of networks in it, along with methodological advances in agent-based modeling in economic research and policy experimentation, from basic agent-based simulation models as narratives in different economic sectors. Therefore, it is considered that the concept of innovation is dynamic and complex and that its study depends on the interaction between the agents that participate in the system at different levels. In this article, the databases of the Global Innovation Index [18] and the Human Development Index [19] were studied to determine the main characteristics that define a country to be in a certain position in the Global Innovation Index. As a result of this analysis, a high correlation was obtained between the dimensions of ranking in technology, level of schooling, and life expectancy statistics to answer the question: What are the principal factors that facilitate a country’s capacity for innovation?

This study proposes to address these gaps by providing an agent-based simulation model that not only integrates these various factors but also allows for a dynamic and predictive understanding of innovation processes. This will facilitate the design of more effective and customized policies to foster innovation in different national and regional contexts.

### Theoretical framework

Schumpeter [20] states that innovation is a major driver of a country’s economic dynamism; the diffusion of an innovation is driven by the aggregate behavior of individuals as well as by interactions between individuals, thus providing a society with a unique social culture through the generation of new innovations. This factor accelerates not only economic growth, but also other factors such as international competitiveness, environmental sustainability, and improvements in a country’s welfare [21].

Wu et al. [22] examine and deepen through various studies the relationship that the influence of financial flexibility on firm performance has or counts on, paying special attention and investigating details how investment efficiency and investment scale can moderate this relationship, so that together they facilitate the work in the financial field. Thus, using a dataset of highly listed companies, the authors found that financial flexibility has a positive impact on firm performance. In addition, this relationship is strengthened in companies with high investment efficiency and working with a higher investment scale, achieving a faster and more efficient adaptation to the conditions that the market presents, and also allowing them to take advantage of the investment opportunities that arise in the process. These findings suggest that it can work on various successes they have found, where we say that companies that efficiently manage their financial resources and have the tendency to invest on a large scale can maximize the benefits of financial flexibility, thus improving their overall performance and obtaining better results for the company along with a significant return on investment and low risk.

Alkaraan et al. [23] conduct an exploration and/or research on how Industry 4.0, green business strategies, and strong governance succeed in strengthening green business strategies that are oriented towards sustainability, because new initiatives that enable the sustainability of companies come from artificial intelligence and the Internet. This research tells us that the vision on which it is based is directed to natural resources, where natural resources are the basis of strategies that are considered sustainable because they contribute to efficiency and generate less environmental impact and the theory of dynamic capacity that talks about having an ease of adaptation in the business environment and the demands of sustainability, this allows having a more dynamic capacity to any impact, the authors study the unification of advanced technologies and governance practices in companies because if you have a consistent and clear governance makes them meet their commitments to sustainability. On the other hand, they found that the adoption of Industry 4.0 technologies is extremely important, such as artificial intelligence and the Internet of Things (IoT), together with effective governance, eloquently enhances the sustainability capabilities of companies. This is achieved through the optimization of resources, the work to be done to improve decision making and the understanding of resources that give the ability to adapt to environmental changes. In conclusion, the results of the study underline the importance of technological innovation and sound innovation governance in the development of business strategies that are not only competitive but also sustainable in the long term.

Alkaraan et al. [24] conduct a study on how the business transformation led by Industry 4.0 manages to significantly impact the financial performance of companies, despite and as a result of the influence of environmental, social and governance (ESG) factors. The study uses and has a quantitative approach that allows the evaluation of how the adoption of advanced technologies impacts companies or their business system depending on the industry in which it applies, such as automation and artificial intelligence, this goes hand in hand with ESG practices considered robust, but it has the facility to improve operational efficiency and profitability. The results show that companies that innovate and integrate Industry 4.0 technologies and maintain high ESG standards tend to have better financial performance and are more competitive in the market, highlighting the importance of a holistic strategy that includes both technological innovation and corporate responsibility.

The article by Alkaraan et al. [25] titled "*A new era in strategic investment decision-making practices in UK companies*: *towards sustainable supply chains and a circular economy*" explores how UK companies are adapting their practices towards more sustainable investment decision-making practices in a way that aligns with sustainability (supply chains) and the circular economy. The study presents the challenges and opportunities that arise as they begin to implement new practices such as Industry 4.0, adding the use of big data, artificial intelligence and advanced analytics in sustainable supply chains, among other factors that positively contribute to change.

The authors emphasize the necessity of studying the interests and expectations of stakeholders to facilitate the development of strategies that enable comprehensive and efficient resource management at the organizational level and for individual stakeholders. In contrast, policies illustrate the manner in which diverse business models have been transformed in accordance with the principles of the circular economy. This transition has yielded discernible benefits, including enhanced efficiency, productivity, operational sustainability, and the comprehensive integration of stakeholders.

Boisier [26], point out that economic growth is the platform for the economic and social development of countries. Its value lies not only in the macroeconomic wealth it represents for a country, but also in the fact that it is the basis from which processes can be generated to optimize the quality of life and living conditions of the population. The decline in the wealth of nations has a negative impact on social welfare, as it reduces the capacity of the state to promote better living conditions for the population [27]. A key component for accelerating economic growth and, consequently, stimulating social development is innovation, as it enhances human and teamwork capabilities among agents, activating the growth and development of countries.

Baumann and Winzar [28] emphasize the necessity of discerning which elements of the educational process require reevaluation to facilitate innovation and the implementation of more experiential and meaningful practices. This, in turn, enables educational communities to embrace new languages and narratives, with significant implications for both human capital and companies, as individuals are trained effectively through education. Vargas Mayo asserts that education must be adapted to enhance the competitiveness of professionals in the contemporary labor market.

Tovar [29] asserts that universities must implement various modifications to their curricula to achieve positive outcomes and align with the demands and objectives of the labor market. He emphasizes the digital environment and the innovation it brings, which enhances the competitiveness of companies by enabling them to integrate highly skilled and well-trained human capital. Conversely, the training of universities should be based on two variables: theory and practice. However, practice should take precedence so that graduates can adapt efficiently and quickly, thereby contributing to the global competitiveness of companies.

In their study, Handel and Hanushek [30] examine the impact of educational quality on economic and business competitiveness, incorporating a range of variables to shed light on this crucial relationship. Their research underscores the necessity of prioritizing the enhancement of cognitive and mathematical abilities as indispensable elements of quality improvement. This is directly correlated with augmented labor productivity, which, in turn, enhances the competitiveness of companies.

García and Heckman [31] have been investigating the long-term impact of investments in education on productivity and competitiveness. They have found that the most effective way to achieve these outcomes is to start education at an early age, as this allows for the development of human capital and the acquisition of labor skills that enhance competitiveness in the workplace.

In recent years, the social aspect of innovation diffusion has attracted increasing attention due to the rapidly changing social environment, especially due to the emergence of new communication technologies. As innovation is considered an emergent and complex phenomenon due to individual interactions, agent-based models have been applied in innovation diffusion studies. Zhang and Vorobeychik [32], thus providing theoretical exploration and support for countries’ public policy decisions.

Sengupta & Sena [33] in their study they develop new models of open innovation (OI) behavior by comparing the impact of two types of OI frameworks: open source (OS) and patent licensing (PL). The dynamic consequences of OI for both OS and PL are studied using a complex adaptive systems approach. In each case, the evolution of profits, technology levels, R&D investments, technology adoption, and market structure are examined and influenced by the underlying market characteristics. While both OS and PL are equivalent in technological performance, OS offers additional advantages for participating firms. Firms in OS earn higher profits and are more efficient in their R&D investments. The industry is less concentrated in OS than in PL, except when the market size is very large.

Paiva, Ribeiro & Coutinho [34] have conducted extensive research on innovation and sustainability in companies. Their studies discuss the importance of making an investment in R&D due to the fact that research and development allows companies to identify different aspects in which they should work and how they should improve the same. It is crucial for companies to retain their competitive advantage, especially in the face of markets and demands that are constantly changing due to technology and globalization.

In a 2015 [35], Klaus Schwab, the executive chairman of the World Economic Forum, discussed the impact of the Fourth Industrial Revolution on business. He emphasized the importance of significant investment and the necessity for human capital to utilize it effectively and efficiently. This, he argued, is crucial for maintaining competitiveness in an environment of rapid technological evolution. In the contemporary globalized environment, innovation has become a critical factor for the survival of a company rather than a potential cause of its demise. In order to remain competitive in this rapidly evolving technological landscape, innovating must become a daily practice, a constant and dynamic process rather than an occasional occurrence. Furthermore, innovation must align with the principles of corporate social responsibility (CSR), as it serves as a means of gaining a competitive advantage for the company while simultaneously benefiting all stakeholders and the environment. Despite the impact of technological advancement on traditional work structures, organizations must prioritize the development of their human capital, which represents their most valuable resource and the source of innovative ideas. Considering the accelerated pace of change in the contemporary world, both organizations and individuals must endeavor to stay ahead of the curve and adopt a mindset of continuous learning to enhance the skills required to navigate the evolving challenges brought about by change [36].

In other studies, Chesbrough [37] defines OI as a paradigm in which firms "*can and should use external and internal ideas and internal and external routes to marke*t" in their efforts to innovate new products and technologies for the market. Businesses such as energy, healthcare, transportation, and finance have fundamentally changed the way innovation takes place, highlighting the importance of collaboration and co-creation in knowledge markets [38, 39].

Lee et al. [40] show in their studies that the innovation process can be supported by efforts even among competing organizations, allowing for cost and process benefits. While empirical studies have examined how innovative firms adopt innovations, the theoretical literature on this topic is limited [33].

The industrial revolutions have been great movements necessary to advance through the digital transformation, but it has shown that when these revolutions happen, there is a great variety of opportunities, but it all depends on the companies to decide whether to adapt to the opportunity and expand or if the company closes definitively, the same way happens with people, because if the company closes, it is the workers of the company who must find a way to adapt by getting a new job that now requires different skills to work.

Alkaraan [41] examines the disparate studies on the current debates surrounding corporate governance. The review encompasses an overview of corporate sustainability, underscoring the progressive significance of integrating sustainable practices into corporate strategies. The author underscores how companies are responding to stakeholder demands for enhanced transparency and social responsibility. By examining these trends, companies are better positioned to engage in policy formulation and decision-making at the highest levels, including management. Furthermore, it underscores the necessity for enhanced collaboration between corporations and governmental entities in a manner that fosters long-term sustainable development.

And Alkaraan [24] examines the impact of the convergence of Industry 4.0 and the circular economy on the transformation of mergers and acquisitions (M&A) strategies. The author examines the factors influencing the integration of advanced technologies and the incorporation of sustainable practices, demonstrating that these can generate substantial synergies, enhancing operational efficiency and reducing environmental impact. The study considers recent M&A cases in various industries in order to provide evidence of how these transactions facilitate the protection of more resilient and sustainable business models. Conversely, it examines and proposes solutions to the challenges and opportunities encountered by companies in each of the novel contexts they navigate, underscoring the necessity for expedient adaptation to technological and regulatory shifts.

Taking into account the models it is believed that organizations should have a hybrid approach and thus achieve better results from different perspectives within the company, giving the opportunity to build capacity, however, these decisions are made by leaders who must be trained for all that the world brings that allows the improvement of your business, these leaders must have the ability to transform and manage uncertainty while motivating and empowering their teams.

Innovation is based on the commercialization and scientific discovery of all those processes that manage to increase productivity, added value and economic logic that allows to focus the market and remain competitive with other companies, thus creating numerous commercial opportunities along with employment opportunities and scientific research as a complete set that allows the improvement of chaos and takes as opportunities the mistakes made in the past.

The National Innovation System prioritizes formality in order to maintain and expand its competitive advantage both domestically and globally. The creation of knowledge that leads to innovation allows for the design of high-quality products, positioning the state as a risk-taker and providing opportunities for growth in new technologies. This is achieved through research, development, and scientific activity.

Technological innovation aims to reduce the environmental costs of economic growth. This will prevent ecological disaster caused by the constant growth of products and services that harm the environment. The transformation and cost reduction can be achieved by stimulating regulatory compliance and economic growth of individual companies.

In 2017 [42], Alkaraan employs a multidisciplinary approach to evaluate strategic investments. They integrate the methodologies of various disciplines to develop arguments and strategies that inform decision-making processes and have a meaningful, long-term impact on the performance of managers at the corporate level. The author underscores that investments generated at the corporate level, contingent on the industry and company in question, including mergers and acquisitions (M&A), frequently entail elevated levels of risk and outcomes that are challenging to quantify.

The study demonstrates how the integration of diverse methodologies, including real options analysis, technology mapping, and benchmarking, facilitates a comprehensive and nuanced understanding of innovative processes and their implications for strategic decision-making. In his 2017 study, Alkaraan underscores the necessity of considering both financial and non-financial elements when evaluating these investments. This approach is designed to maximize corporate value and ensure successful integration.

The goal of environmental care is to promote moderate and natural consumption and production of resources that drive the economy, to prevent destructive effects on our planet. It is not only the responsibility of companies to make changes, but also of individuals to adopt more sustainable patterns of consumption and production. Therefore, we must raise awareness to increase resource efficiency and promote a sustainable lifestyle. A sustainable lifestyle also indirectly contributes to reducing poverty and achieving a transition to green economies with lower carbon emissions. During the COVID-19 pandemic, it has been emphasized that people should maintain a clean, limited, and respectful relationship with nature to avoid negative impacts on the planet. This is reflected in consumption and production through sustainability [43].

Society has expressed concern over the lack of social, cultural, and environmental responsibility demonstrated by companies. This has led to both non-violent and violent demonstrations due to the limited changes that have occurred in the digital era. Therefore, it is important to incorporate indicators that can measure the well-being of individuals on a large scale and track daily changes.

The COVID-19 pandemic has prompted changes in our behavior towards the environment, as we have reduced our consumption and improper use of nature. This has led to an increase in sustainability among companies, resulting in the development of tangible metrics that measure personal income, wealth, security, and welfare on both macroeconomic and microeconomic scales. These metrics evaluate the fulfillment of basic needs for individuals.

On the other hand, each of the reforms is directed towards improving the quality of education and promoting innovation. This is because there is a correlation between financial resources and student performance. Students are the new generation that will restructure and change the inequalities that exist, and they are also responsible for maintaining sustainable land for future generations [44].

Changes are initiated by individuals through their consumption choices. However, a deficient educational system can perpetuate a vicious cycle of crime, poverty, and inequality. Therefore, a comprehensive education is necessary to break this cycle. Therefore, the innovative mechanisms aim to improve educational outcomes while recognizing the need for an equitable process for all students involved. However, achieving equity and equality within educational institutions requires structural changes in public education regulations. Additionally, changes have been made to the budgetary policy to define assertive financing for the education and training of new generations.

Countries that have developed capabilities to produce and use existing knowledge have technological and industrial advantages. This allows them to create designs, manufacture new tools, commercialize goods, and provide services that positively affect the economic growth and social development of the nation [45]. As the educational system and trends have evolved, technology has played a crucial role in achieving two main objectives: determining the relationship between information technologies and innovation capacity and identifying and integrating innovation and technology through the admission and placement test process. The educational system aims to train and educate in multiple dimensions, including quality leadership, management, staff interaction, productivity of the institute, and control and measurement of processes. When it comes to innovation, it is important to learn during the process and reinforce that knowledge, which allows for the development of sustainable competitive advantages.

Rincón-Soto, Rengifo-Lozano, Hernández-Suárez & Prada [46] emphasize the importance of education in the development of nations. They suggest that promoting variables such as management, entrepreneurship, and innovation are crucial for sustaining economic growth, overall development, and human development. This is the ultimate goal of any public policy. Therefore, it is vital to constantly review educational systems and their progress, especially in the face of the volatile and complex scenarios present in the world economy.

Simionescu et al. [47] conducted a series of studies examining the relationship between economic growth and income per capita. Their findings consistently indicate that when income per capita is high, it is associated with a number of positive outcomes. It is reasonable to assume that a higher demand will be observed, given that goods and services of high quality will be demanded. This will facilitate and impel companies to improve and become more competitive, offering products and services of quality that will act as a competitive advantage on a global scale. This will enable them to satisfy the needs of the market, despite the high expectations that will be placed upon them.

### Similar studies

Agent-based models (ABMs) are computer simulations used to model the behavior of a finite collection of interacting individuals or agents. These agents have unique attributes related to spatial location, physiological traits, and/or social behavior. ABMs work bottom-up, with population-level behavior arising from interactions between autonomous individuals and their environment. The history of each individual can be traced, and network structures can be explicitly represented. In general, agent-based models (ABMs) enable: (i) The aim is to introduce local interaction rules at the individual level that closely match physical and social interaction rules. (ii) Additionally, behaviors that can be randomized at the observational level but are deterministic from a mathematical point of view should be included. (iii) A modular structure should be incorporated, and information should be aggregated through new types of individuals or by modifying existing rules. (iv) Lastly, observe system dynamics that could not be inferred from examining the rules of individual participants.

Roozmand et al. [48] simulated consumers’ decision-making process for automobile purchases based on power distance and personality, using three automobile characteristics: social status, social responsibility, and price. The utility of an individual depends on their level of power distance, social status, and personality.

Railsback & Grimm [49] introduced an agent-based model to investigate individuals’ movie selection decisions. In their model, an individual’s decision is probabilistic based on their utility, which is a weighted sum of individual and social utility. Social utility is divided into two separate social influences: the influence of the past behavior of other strangers and the influence of the individual’s social network. Additionally, the authors analyze the differences in social influence in different cultures based on individualism.

Ning et al., [50] propose an agent-based model for electric vehicle adoption in Germany. The social network structure is determined by individual characteristics such as age, gender, income, education, place of residence, lifestyle, and social radius also consider socioeconomic factors when generating a small-world social network in their agent-based model for solar PV system diffusion in Italy. They adjust the probability of connection between and within different social groups based on adoption categories.

## Methodology

This article uses a quantitative-correlational methodology to construct an agent-based model for the purpose of identifying the determinants of innovation in American and European countries. Two databases were utilized in this study: the Global Innovation Index [18] and the Human Development Index [19]. A detailed description of the data and its constituent indicators was provided.

Subsequently, the data were analyzed to identify the variables with the strongest association with the Global Innovation Index. The methods employed were Spearman’s nonparametric correlations and multiple hierarchical regressions.

Once the principal variables associated with the Global Innovation Index were identified, the ODD (Overview, Design, Concepts and Details) methodology was employed. This is a structured framework for describing multi-agent systems [51, 52]. This methodology ensures that the models are described in a coherent and complete manner, thus facilitating comprehension and reproduction of the results by other researchers.

Finally, an experimental design was conducted to ascertain whether the simulation could reproduce the global innovation index based on the defined rules. A correlation analysis was employed to determine the relationship between the values generated by the model and those of the global innovation index. The following sections describe each step of the methodology.

### Global innovation index

The Global Innovation Index [2] is used as an input to determine a country’s level of innovation. This index helps identify leading innovation countries and provides a framework for countries to develop and improve their innovation policies. It consists of seven pillars and is a key predictor of a country’s innovative capacity.

#### 1. Institutions

**Fig 1:**
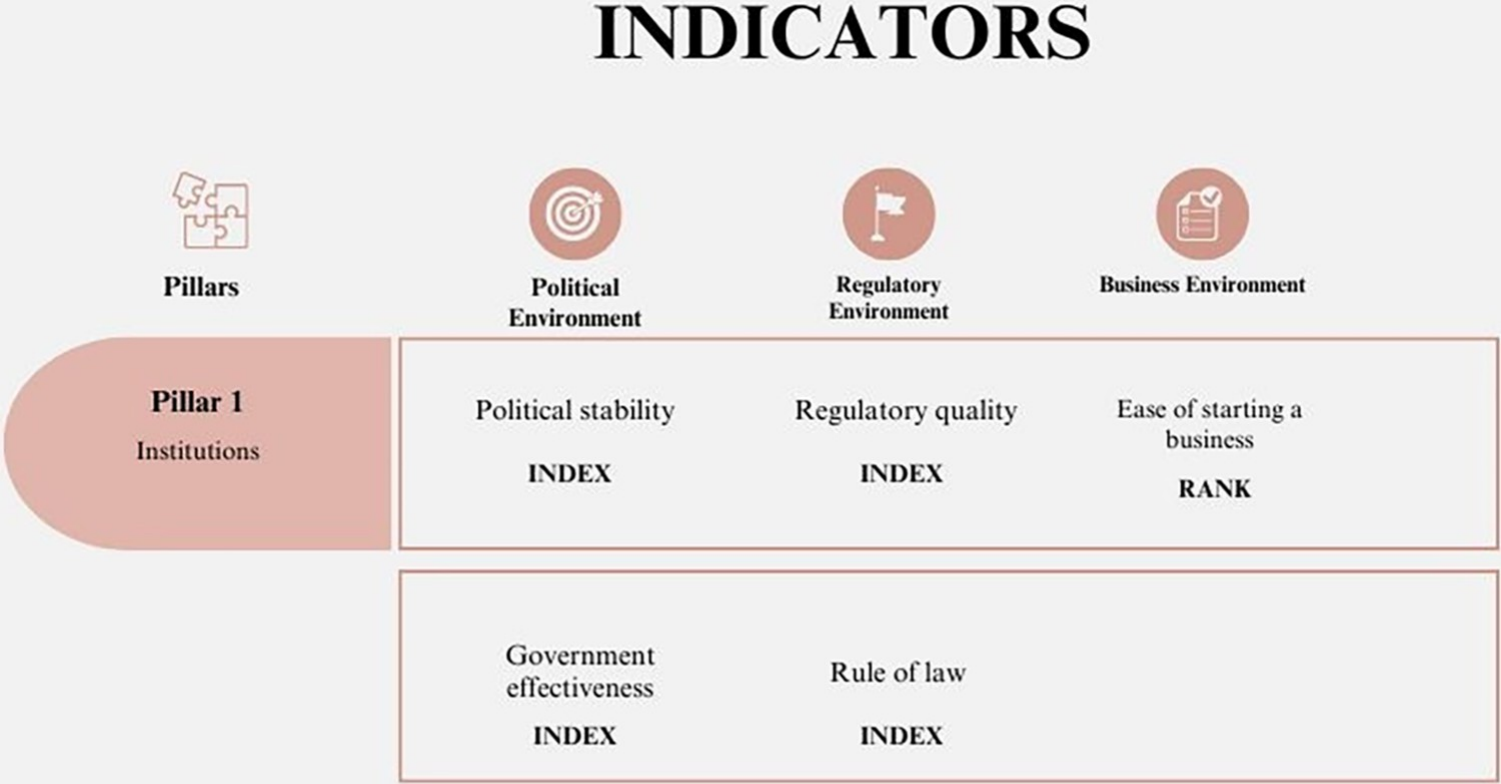
Pillar 1 –Institutions. Source: Own elaboration.

Economies require a strong productive apparatus, and institutions play an essential role in generating norms and establishing favorable environments for business development. Thus, actors in economies consider economic, social, political, and environmental factors when making decisions. This is based on the interaction of various structures that contribute to the organization of society and the business community [53]. A summary of the Institutions Pillar is shown in Fig 1.

#### 2. Human capital and research

**Fig 2:**
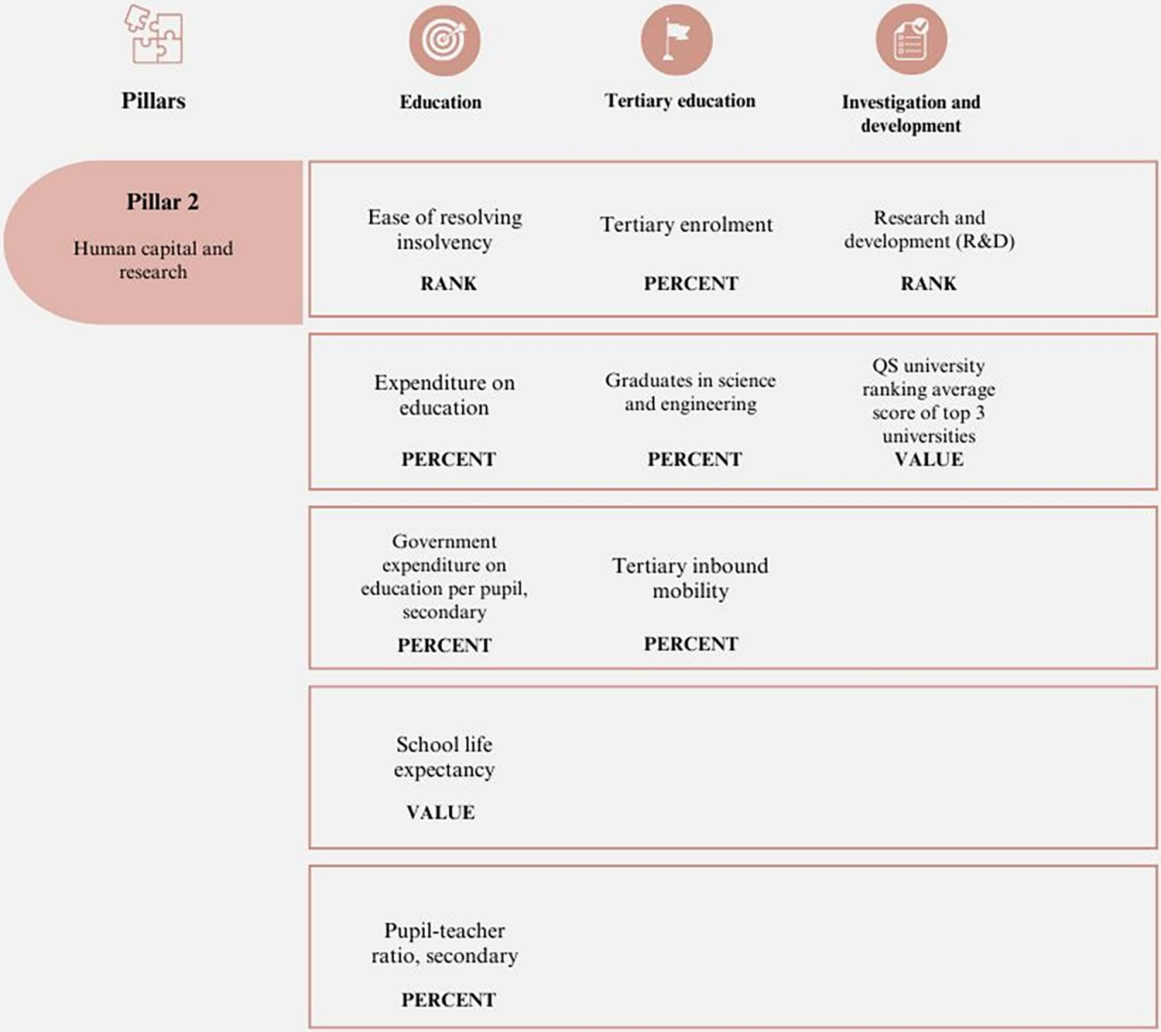
Pillar 2 –Human capital and research. Source: Own elaboration.

At the economic level, human capital is recognized as a significant determinant of innovation. The literature on the value of innovation highlights the importance of highly qualified human capital for fostering an innovative culture within a company [54, 55]. Therefore, science and technology can help reduce social gaps. Efforts are being made in the Western world to generate a culture of innovation that is sensitive to demands and integrates citizens into the processes of research and development. As shown in Fig 2, this requires a nexus between science, technology, government and society.

#### 3. Infrastructure

**Fig 3:**
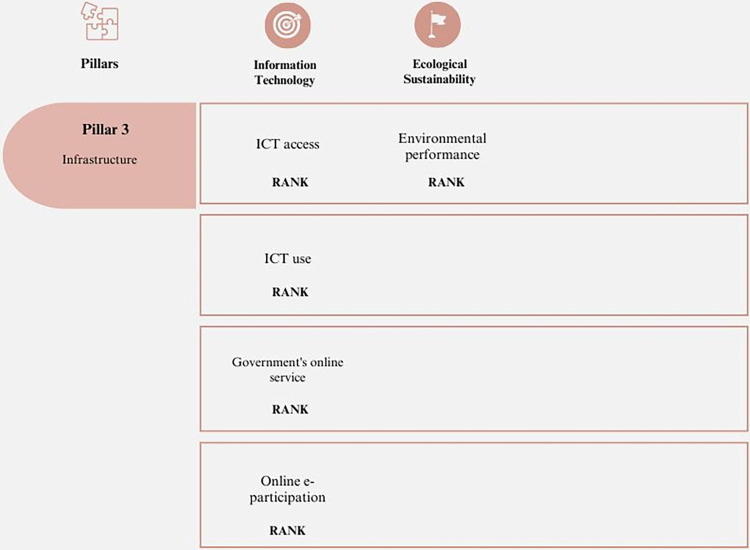
Pillar 3 –Infrastructure. Source: Own elaboration.

Measuring innovation involves considering the resources used by countries and companies to increase productivity and economic growth [56]. Physical infrastructure, ICTs, and resources that facilitate business sustainability are important for incorporating concepts that enhance the activity of companies, sectors, and economies. Projects that significantly impact management can be developed with these resources [57]. Fig 3 provides a summary of this pillar.

#### 4. Market sophistication

**Fig 4:**
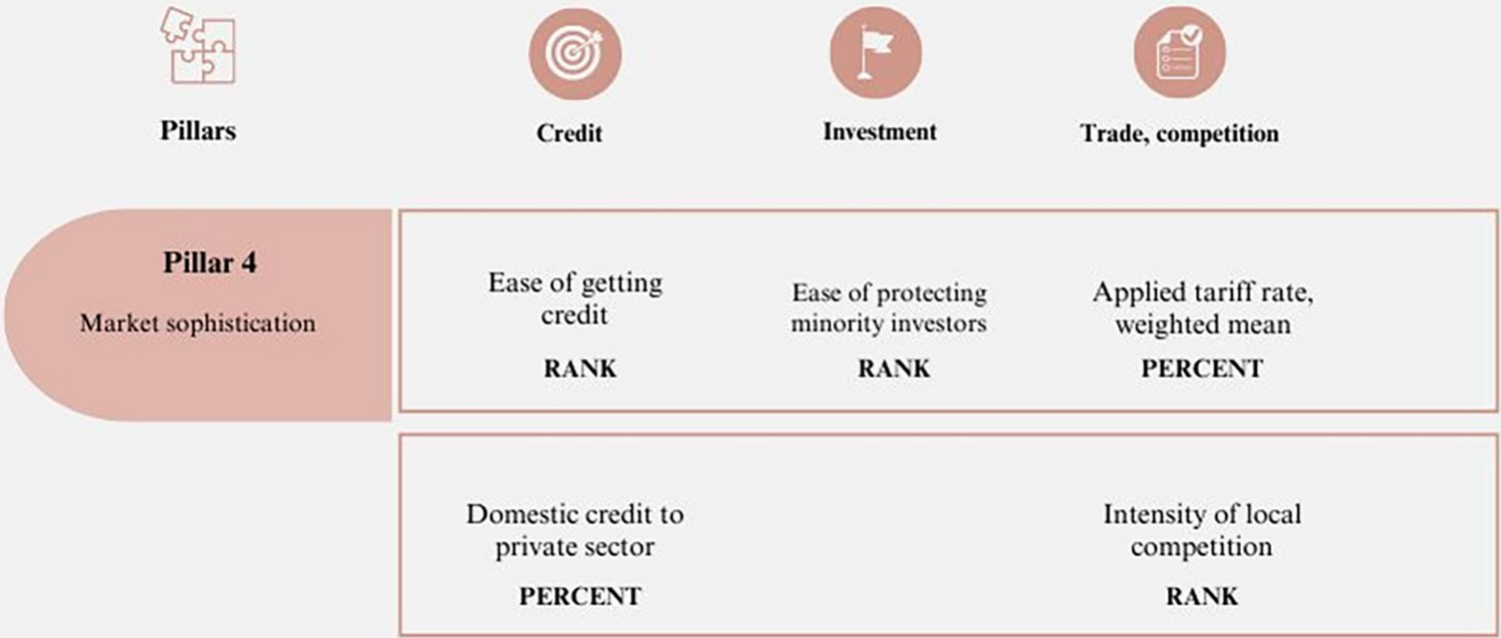
Pillar 4 –Market sophistication. Source: Own elaboration.

Although market sophistication is an essential component for competitiveness, its role in consolidating companies and sectors of an economy lies in identifying market segments, characterizing customers, and recognizing products and trends. When business ecosystems identify competitive advantages, they can position themselves to favor economic growth by developing and interacting with the needs that arise in the market. This requires the incorporation of elements related to innovation, technology, and new marketing trends, among others [58]. These elements are summarized in Fig 4.

#### 5. Business sophistication

**Fig 5:**
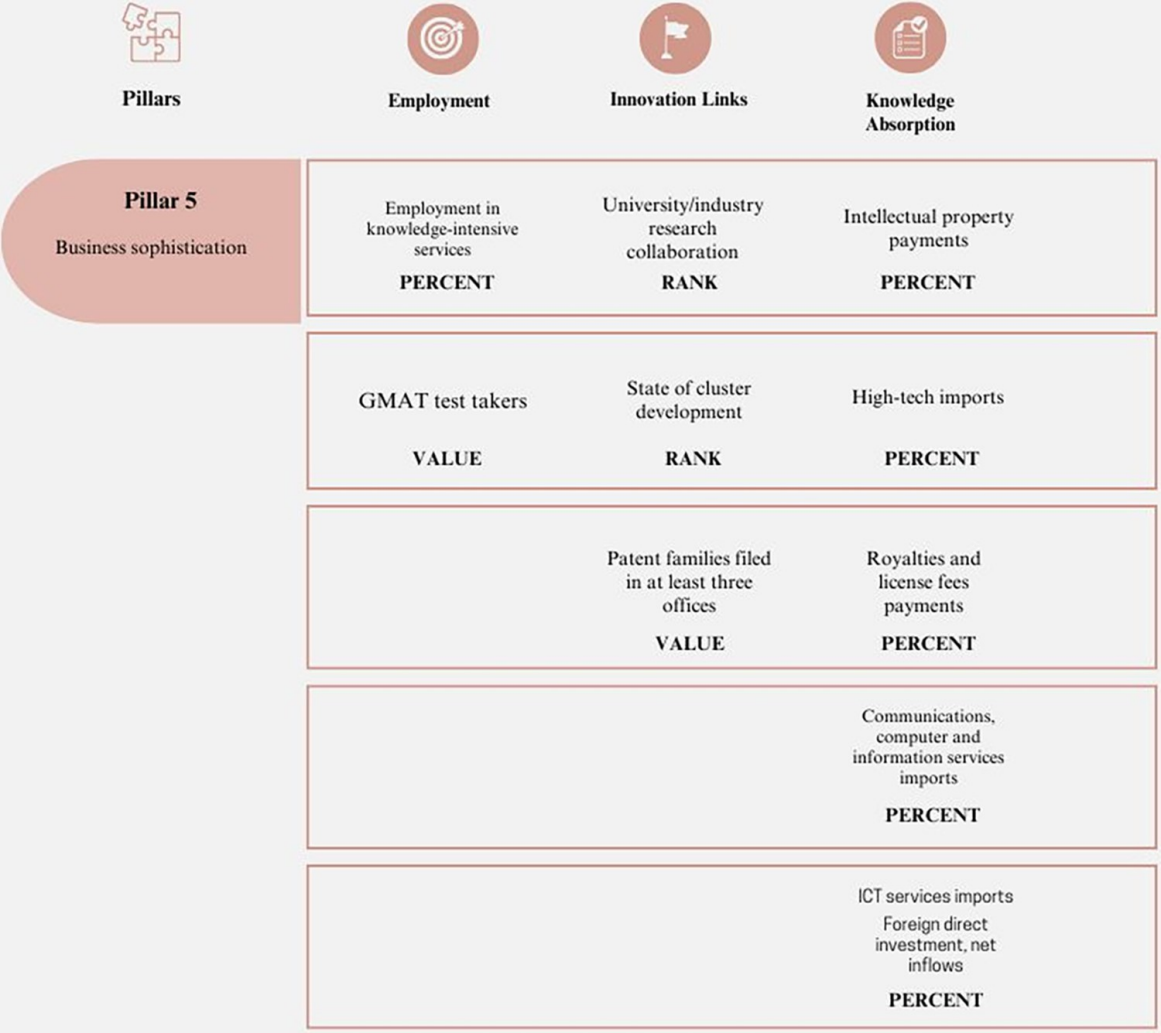
Pillar 5 –Business sophistication. Source: Own elaboration.

The growth of economies and sectors is significantly influenced by the relationships between actors. To achieve this, it is essential to incorporate strategies and policies that promote the development of structured and competitive businesses [59]. Business sophistication is determined by networks, strategies, quality of operations, and collaborative work. This knowledge can contribute to economic growth. Fig 5 illustrates the Business Sophistication pillar.

#### 6. Knowledge and technology outputs

**Fig 6:**
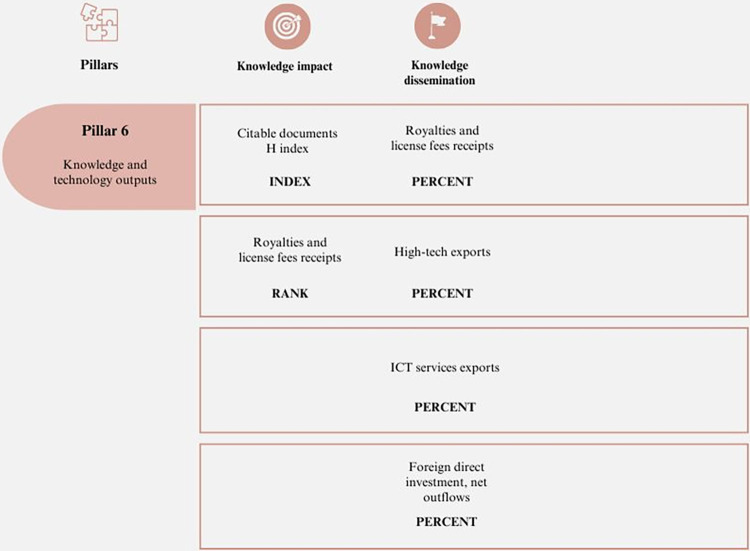
Pillar 6 –Knowledge and technology outputs. Source: Own elaboration.

The implementation of technological processes is crucial for the development of productive activities and the increase of their economic potential. Additionally, it significantly contributes to the improvement of various areas and the generation of competitive processes [60]. Fig 6 summarizes this pillar.

#### 7. Creative outputs

**Fig 7:**
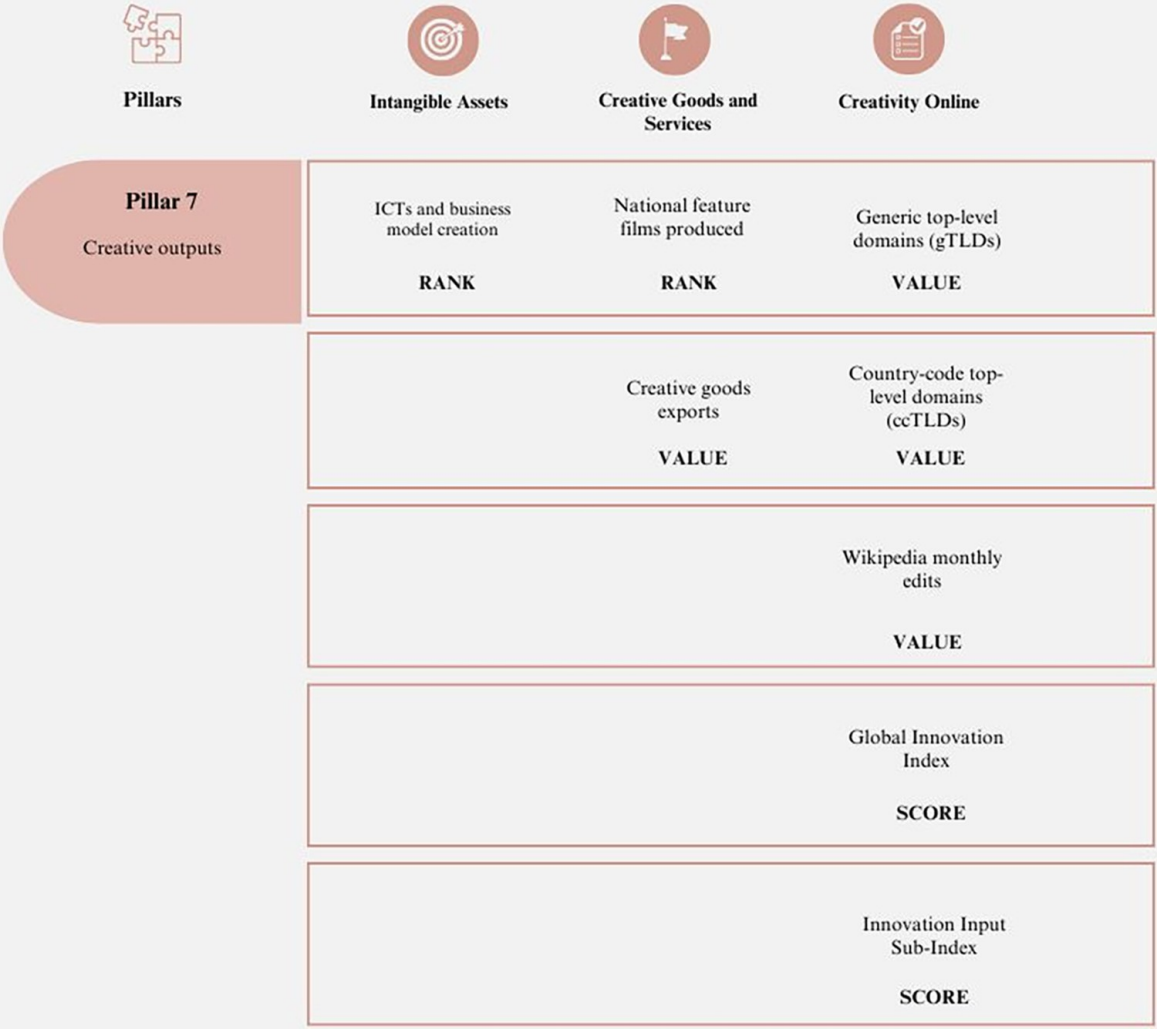
Pillar 7 –Creative outputs. Source: Own elaboration.

This sub-index pertains to the development of processes that promote a creative culture within the organization and its productive growth. These processes also play a significant role in creating a competitive culture by implementing activities that aim to position products, improve services, and change the mindset of market participants [61]. Fig 7 introduces the key aspects of this pillar.

### Human development index

**Fig 8:**
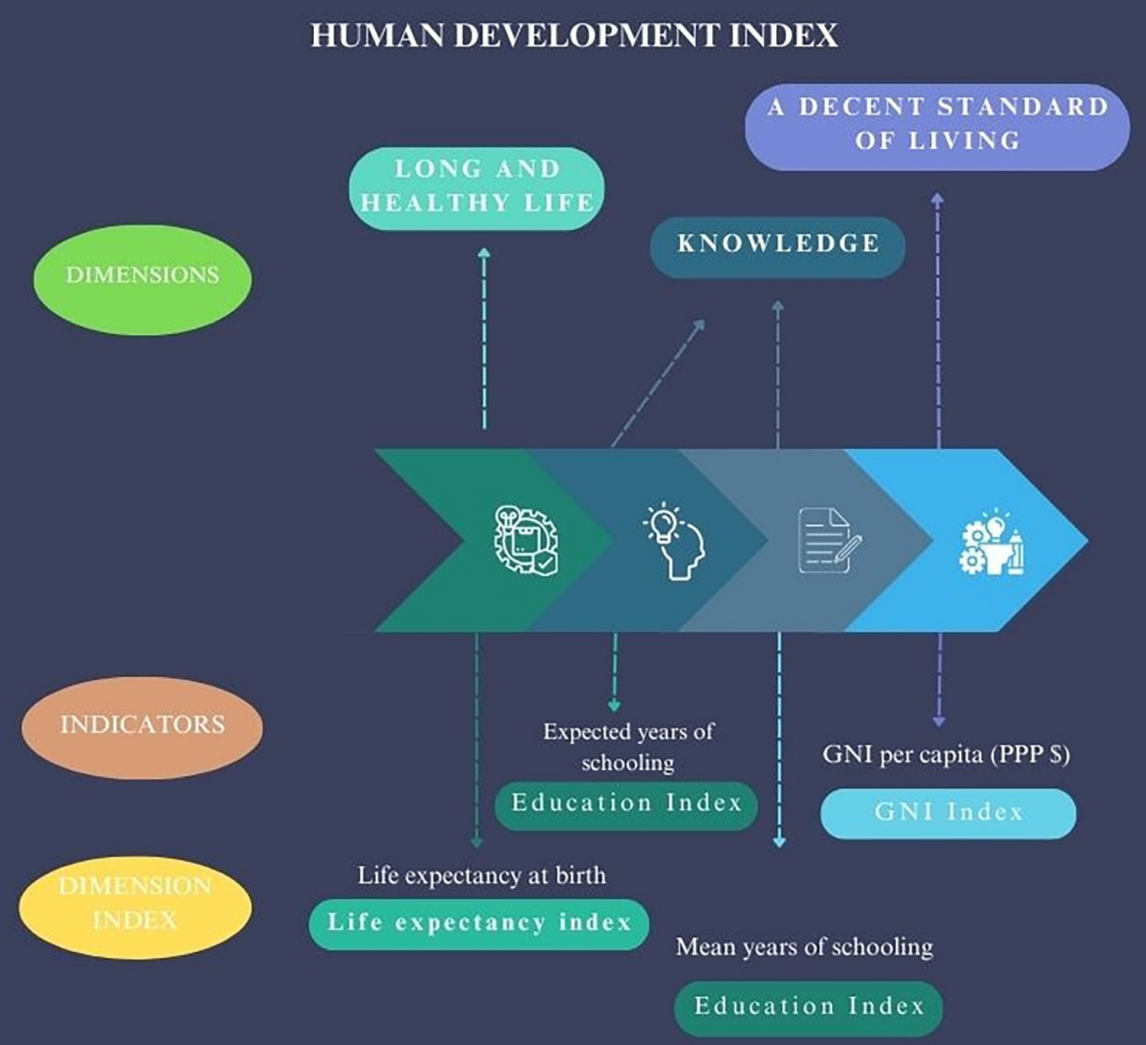
Human development index. Source: Own elaboration.

The *Human Development Index (HDI)* database is also utilized in this study [14]. The Human Development Index (HDI) is a set of indicators that measure a country’s level of development. The HDI includes parameters that go beyond economic income. In 2020, a new variable related to carbon dioxide emissions was added. The following indicators will be used to measure various aspects of human development: life expectancy at birth, inequality in life expectancy, maternal mortality rate, adolescent birth rate, expected years of schooling, average years of schooling, percentage of people with secondary education, inequality in education, gender development index, gross national income per capita (GNI), income inequality, human inequality coefficient, gender inequality index, percentage of women in parliaments, and participation of men and women in the labor market. Additionally, a multidimensional poverty figure will be calculated. From the human development perspective, well-being is linked to people’s capabilities rather than their material wealth. To lead a dignified life, individuals require a range of capabilities that enable them to fulfill basic needs, such as food, education, transportation, and the exercise of political rights, among others [62]. The dimensions, indicators and dimension index of the HDI are shown in Fig 8.

### Data analysis

To initially analyze the collected data, SPSS software [63] was used. The analyses conducted were Spearman´s nonparametric correlations and multiple hierarchical regressions. For the particular case of this research, these analyses had the purpose to identify those variables with the highest association with the dependent variable (global innovation index), which later were used for the model of agents. Also, the regression analyses gave us insight about the specific contribution of the selected variables when using them for the constructed model, as well as multicollinearity among them.

Concerning the correlation analyses conducted for this research, the data reported in the Global Innovation Index from years 2015 to 2020 were used. Additionally, the Human Development Index results from 2014 to 2019 were also analyzed, specifically, considering data from life expectancy at birth, expected years of schooling, mean years of schooling, and Gross National Income (GNI) per capita. As it was mentioned before, the analyses were conducted in order to identify those variables with the highest relations with the Global Innovation Index score.

For the data from the Global Innovation Index report, five variables were found to have relations above .8 across all the years analyzed. These variables were ICT access, ICT use, Innovation input sub-index, Innovation output sub-index and Research & Development. According to this result, and with the purpose to avoid redundancies among the aspects covered by these variables, ICT use and Research & Development were selected to further regression analyses. Regarding the data from the Human Development Index, the correlation analyses indicate coefficients above .8 on Mean years of schooling, GNI per capita and Life expectancy at birth. According to these results, the three variables were also selected for the regression analyses. The results of hierarchical multiple regressions performed with the selected variables from the correlation analyses are presented in Table 1.

**Table 1:** Hierarchical multiple regressions results.

	Coefficients				Collineality statistics
Model 1	B	Standard Error	t	Sig.	VIF
(Constant)	-15,209	10,103	-1,505	,135	
Mean years of schooling 2019	,961	,308	3,117	,002	2,461
Gross national income (GNI) per capita 2019	,000	,000	5,505	,000	2,438
Life expectancy at birth 2019	,491	,158	3,107	,002	3,037
Model 2					
(Constant)	21,384	13,981	1,530	,129	
Mean years of schooling 2019	,327	,341	,958	,341	3,367
Gross national income (GNI) per capita 2019	,000	,000	2,459	,016	3,849
Life expectancy at birth 2019	,252	,164	1,543	,126	3,638
ICT use 2019	-,157	,044	-3,586	,001	7,934
Model 3					
(Constant)	33,385	11,198	2,981	,004	
Mean years of schooling 2019	,458	,271	1,690	,094	3,380
Gross national income (GNI) per capita 2019	,000	,000	2,560	,012	3,867
Life expectancy at birth 2019	,129	,131	,987	,326	3,693
ICT use 2019	-,059	,037	-1,589	,115	8,994
Research and development (R&D) 2019	-,155	,020	-7,741	,000	2,582

**Note:** Dependent Variable: Global Innovation Index; Model 1: R^2^: .744; Model 2: R^2^: .773; Model 3: R^2^: .859; Durbin Watson: 1.949.

As it can be seen from the regression analyses, the final model tested explained the 85.9% of the dependent variable variance, with acceptable collineality statistics for each of the variables considered. The analysis also indicates that Research & Development and GNI per capita are the most important variables for the model tested (t: -7.741 and 2.56, respectively; p<.01).

### Agent based model

The ODD protocol is used to describe the agent model [52]. The description includes the purpose of the simulation, an overview of the model, some design assumptions, and finally the model details.

### Purpose

The purpose of the model is to examine the impact of gross national income per capita, birth rate, life expectancy at birth, mean years of schooling, research and development ranking and ICT Use Ranking (information and communication technology) use on a country’s Global Innovation Index.

### Entities, states, variables and scales

The model defines two types of entities: country and person. At the country level, the following variables are included:

- Birth rate: Defined as the number of people born each year per 1000 people in the population. A birth rate of 15 means that 15 new people are born each year per 1000 people [64].
- Life-expectancy: age in years defined as a numerical variable to measure the health dimension of the HDI [19].
- Mean years of schooling: Numeric variable measuring the educational dimension for adults aged 25 [19].
- Space: Area in which individuals are distributed, defined as a toroidal space.
- ICT use is a ranking derived from a composite index that assigns 25 percent to each of the following variables—Global Innovation Index Appendix III Sources and definitions [18].
  ○ Percentage of individuals using the Internet
  ○ Wired broadband internet subscriptions per 100 inhabitants.
  ○ Active mobile broadband Internet subscriptions per 100 inhabitants
  ○ Mobile broadband internet traffic (gigabytes/subscriptions)
- Research & Development: Country ranking in terms of research. The ranking considers the number of researchers per million inhabitants, number of Postdocs working in R&D—Global Innovation Index Appendix III Sources and definitions [18].

Each individual has:

- Age: age of the individual in years.
- Years of schooling: integer value that represents the number of years that an individual studies.
- Utility: models the income of an individual. It is a value obtained from the years of schooling of each individual.

### Process overview

The simulation time is defined by discrete rounds, where one round represents one year. The output of the simulation is an estimate of the Global Innovation Index.

At the start of the simulation, the following actions are carried out:

- The simulation starts creating 1000 individuals.
- Initialize the age of individuals with an integer random value between 0 and the life expectancy of a given country.
- Create a value called maximum-years-scolarity for individual.

At the beginning of each round the following process is carried out:

1. Create new people according to the birth rate. Creating a person also involves generating two individual values for each person:
  - Life expectancy: a pseudo-random number generated from 0 to the life expectancy of people of a given country.
  - Maximum years of schooling: a Poisson random number generated from the mean years of schooling of a country.
2. Apply the mortality rate to random individuals: This is done using a Gompertz cumulative density function [65]. The mortality rate at age x is given by
$$\mu (x)\;=\;\alpha e^{\beta x}$$
  Where α and β are constants that are applied using the 50% point of the Gompertz cumulative density function to map life expectancy to the probability of death
$$\alpha =\frac{0.085*0.5}{e^{0.085*lifeexp}-1}$$ (1)
  The actuarial aging rate, β, determines the rate at which the mortality rate increases with additional years. The values of α and β are taken from Netlogo’s Human Population Dynamics model [66]. If a random number is less than μ(x), an individual is removed from the simulation due to death.
3. Increase the age of each individual in one.
4. Evaluate each individual: This step contains to steps:
  - Evaluate Scholarity: Increase the years of schooling in one if the value is less than the maximum individual years of schooling.
  - Evaluate individual incomes: We assume that a person has an individual utility if the age is between 15 and 65. We performed a regression to determine individual utility from individual years of schooling. We obtain the following expression for the income of individual i:
$$utility_{i}=schoolyears_{i}*0.665+4.538$$
5. Update the Global Innovation Index using the expression obtained from the regression. The equation for updating the simulated global innovation index of a country c is as follows:
$$simulatedgni_{c}\;=\;(0.000104*utility_{c})+(lifeexpectancy_{c}*0.129)+(computedav{gschooling}_{c}*0.458)+(ICTUse*-0.059)+(Research\&Development*-0.155)$$
  Where:
  - *utility*_*c*_: is the average utility of country c obtained by all the live individuals.
  - *lifeexpectancy*_*c*_: life expectancy by a country c.
  - *computedavgschooling*_*c*_: is the average utility of country c obtained by all the live individuals.
  - *ICTUse*: ranking obtained from database in Global Innovation Index Appendix III Sources and definitions [13]
  - *Research*&*Development*: ranking obtained from database in Global Innovation Index Appendix III Sources and definitions [13]

### Experiments and results

An experiment is defined with the following input parameters: Birth rate, life-expectancy, mean years of schooling, ICT Use and Research and Development Rank. Data from the year 2019 is used to initialize each experiment. Table 2 shows the sources of information for each parameter.

**Table 2:** Parameter definition for each country.

*Parameter*	*Values of reference/source*
*Birth rate*	Field listing–Birth rate [67]
*life-expectancy*	Human Development Index (HDI) [19]
*Mean years of schooling*	Human Development Index (HDI) [19]
*ICT Use*	Global Innovation Index Appendix III Sources and definitions [18]
*Research and development rank*	Global Innovation Index Appendix III Sources and definitions [18]

Thirty countries were selected for the experiments. The top eleven countries with the highest global innovation index, some countries of Europe and some countries of Latin America. The last country selected was from Latin America, it was number 108 among 186 countries with complete data.

Each experiment was run 30 times for 50 rounds. For each experiment, the last Global Innovation Index obtained is taken as the output of the simulation to simulate population dynamics. The output of the experiment is the 30 estimates of the global innovation index of each country.

**Fig 9:**
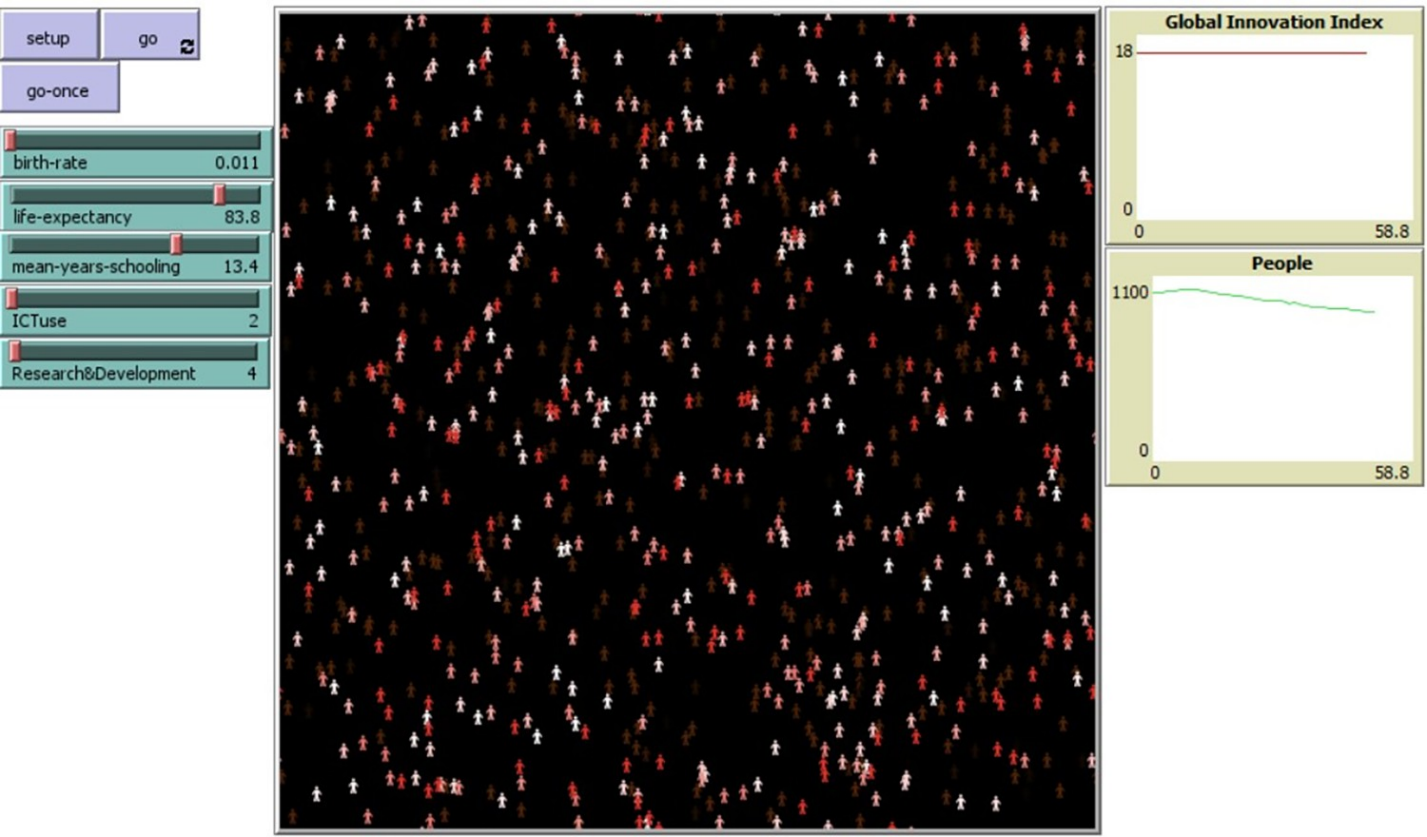
Input parameters of simulation for Switzerland. Screenshot of simulation implemented in Netlogo [68]. Source: Own elaboration.

Fig 9 shows one simulation execution for Switzerland. The simulation has been implemented in Net Logo [68]. Input parameters are defined as sliders on the left. The simulation starts as described in the Process Overview section. Different individual colors represent different ages. Charts at the right are the Global Innovation Index and the population size of a country.

As a final step, a new non-parametric correlation analysis were conducted in order to test associations among the results obtained on the agent experiments and the Global Innovation Index (GNI) scores. To this end, the results obtained in the tested model from 30 Americas and Europe countries, in 30 iterations, were compared with their respective GNI scores from 2017 to 2019. The results showed correlation indexes above .9 with R^2^ indexes above .8 in all cases, indicating a significant association between the predicted results and the actual reported scores (p < .001).

## Discussion

The findings of this study indicate that the variables most strongly correlated with the overall innovation index are primarily associated with average years of schooling, life expectancy at birth, and research and development statistics. These variables were modeled using an agent-based simulation. The simulation results align with the global innovation index, exhibiting a high degree of correlation. This demonstrates that by simulating solely the variables with the most significant impact, a result analogous to that of the global innovation index can be achieved.

The central research question is: What are the principal factors that facilitate a country’s capacity for innovation? One of the most relevant factors that enabled us to ascertain the model is education. In the literature, we find authors who corroborate this finding, including Baumann & Winzar [28], Tovar [29], Handel & Hanushek [30], and Rincón-Soto et al., [46]. The authors posit that education must adapt to new languages and narratives in order to effectively train professionals, thereby enhancing their competitiveness in the contemporary labor market. It is imperative that university education integrates both theoretical and practical variables, with a particular emphasis on the latter. This is because graduates must be able to adapt effectively and quickly in order to contribute to the global competitiveness of companies. It is therefore evident that educational quality has a direct impact on economic and professional competitiveness. Furthermore, variables such as management, entrepreneurship, and innovation are of paramount importance for the sustenance of economic growth, integral development, and human progress, which represents the ultimate objective of any public policy. It is therefore essential to conduct ongoing reviews of educational systems and their evolution, particularly in light of the inherent volatility and complexity of the global economic environment.

The model also indicates the strategic importance of technology. The literature contains numerous authors who support this finding. For example, Canizales-Muñoz [36], Alkaraan, et al., [24] demonstrate that companies that innovate and integrate Industry 4.0 technologies maintain high standards, achieve better financial results, and are more competitive in the market. These authors emphasize the importance of a holistic strategy that includes technological innovation and corporate responsibility. In the context of a globalized environment, innovation is a crucial factor for business survival, and it must be approached as a dynamic and continuous process. It is incumbent upon organizations to cultivate and reinforce their human capital, which is the most valuable asset and a perpetual source of innovative concepts. In light of the accelerated pace of global change, it is imperative for organizations and individuals to adopt a mindset of continuous learning and adaptation. This entails developing the skills necessary to navigate the evolving landscape and meet the challenges posed by change.

In regard to the per capita versus competitiveness variable, additional findings are revealed that serve to reinforce the initial results. Simionescu et al., [47] their results demonstrate that when a country has a high per capita income, it can access a greater number of resources and invest in technology to improve the supply to the market. This is because a higher income allows access to a higher level of innovation and development in companies, which increases competitiveness and demand. Barro [69] posited that: Barro, an economist at Harvard University, has conducted several studies on economic growth and per capita income. In each case, he reached a general conclusion, namely that a high per capita income results in higher demand, which in turn facilitates and encourages companies to improve, become more competitive, and offer quality products and services that provide a competitive advantage globally. This is done in order to satisfy the needs of the market; despite the high expectations it demands. The following section will examine the dimensions of access to technology, education, and life expectancy.

### Access to technology

In accordance with the tenets of innovation theory, the availability of novel technologies can serve to expedite the process of innovation within organizational frameworks. This is achieved by enabling the generation of novel concepts and solutions. It can be reasonably deduced that nations with greater access to sophisticated technology are more likely to develop and implement innovations.

Access to technology has the potential to transform entire sectors, improving efficiency and opening up new possibilities for the creation or improvement of products and services. Countries with a robust technological infrastructure, such as access to high-speed Internet and emerging technologies such as artificial intelligence, tend to lead in technological innovations.

Future research could focus on how different levels of access to technology specifically impact different industries and how technological barriers affect emerging versus advanced economies. In addition, the role of government policy in improving access to key technologies could be investigated.

### Education

Education is a fundamental element in the development of innovative capabilities. It is not only a means of acquiring technical skills and knowledge, but also of fostering creativity and critical thinking, which are essential for innovation.

A well-educated workforce is a prerequisite for innovation. It can be observed that countries which have robust and easily accessible education systems tend to have a population which is better prepared to create new technologies and solutions. Moreover, higher education and research are indispensable for the development of new technologies and knowledge.

Areas for Further Research: It would be beneficial to examine the impact of disparate education systems and investment policies on a country’s capacity for innovation. Furthermore, it would be beneficial to examine the extent to which technical and vocational education contributes to innovation in comparison to general education.

### Life expectancy

The life expectancy of a population can influences the rate of innovation within that population. This is due to the fact that a longer life expectancy allows for a greater overall well-being of the population, which in turn allows for a greater ability to contribute to innovation. A longer life expectancy is associated with superior overall health and an enhanced capacity of individuals to contribute to the workforce and pursue learning opportunities throughout their lifespan. Furthermore, a population that is both healthy and long-lived can sustain an active and creative workforce for a longer period.

It can be observed that countries with a high life expectancy tend to allocate a greater proportion of their resources to the public health and well-being of their population. This can result in the development of a more productive and creative workforce. Furthermore, a favorable state of health can facilitate greater involvement in educational pursuits and the advancement of novel technologies.

As a topic for future research, it would be beneficial to investigate the manner in which life expectancy interacts with other factors, such as access to education and technology, to influence innovation. Furthermore, it would be beneficial to examine the relationship between public health policies and population aging and their impact on a country’s innovative potential.

The conjunction of these factors demonstrates that the nexus of technology, education, and life expectancy engenders an ecosystem conducive to innovation. Each factor reinforces the others, thereby creating a virtuous circle of progress. Countries that invest in technology, education, and health can establish an environment more conducive to innovation. The synergy between these elements can lead to greater competitiveness and economic growth.

## Conclusions

This article puts forth an agent-based model, designated as Innovameter, with the objective of elucidating the underlying factors that influence innovation in disparate contexts, such as those observed in the United States and Europe. A data analysis was conducted to ascertain the primary variables associated with not only the data points of the global index, but also with variables pertaining to the Human Development Index and its impact. The variables identified through regression analysis, including average schooling, GNI per capita, life expectancy at birth, the use of information technologies, and a ranking of research and development, have been demonstrated to be pivotal determinants in the formation of national levels of innovation.

The findings of this study demonstrate that the agent-based simulation model, Innovameter, is capable of accurately reproducing the observed trends in the Global Innovation Index. By simulating the interactions between these variables at the individual and national levels, Innovameter offers a promising approach to understanding the complex interplay of factors that foster innovation in different countries. Further research and experimentation with this model may provide valuable insights for policymakers seeking to design effective strategies to boost innovation and economic growth.

From this initial model, as a possible future work, it is intended to model at the individual level the local rules that allow determining the use of technology and the research and development metrics. In addition, the parameterization of the models could be used to identify aspects or policies that could be adopted for the development of innovation policies in a country.

The data used in the development of the article, the results of the experiments, and the source code are available at the following link: https://github.com/arleserp/Innovameter

### Theoretical and practical implications

From a theoretical standpoint, our findings indicate that five variables demonstrate relationships exceeding 0. In the eight years under examination, the model developed in this research was able to examine the impact of six variables on a country’s overall innovation: gross national income per capita, birth rate, life expectancy at birth, average years of schooling, research and development ranking, and information and communication technology (ICT) use. The model also provides data to assess the impact and effectiveness of existing policies. This allows for adjustments and improvements to be made, thereby ensuring that resources are used as effectively as possible.

The model can be utilized to investigate novel avenues of inquiry into the factors that influence innovation in countries, thereby enabling governments to develop policies that will facilitate improvements in their innovation performance.

In conclusion, the models proposed here can be utilized by business administration professors to instruct students in the various strategies that can facilitate innovation within organizations. Moreover, students can be tasked with analyzing the manner in which each factor exerts a beneficial influence on a company’s capacity for innovation.

### Limitations of the study

The model developed in this paper is designed to simulate the dynamics of life expectancy, schooling, and income by representing agents that emulate individuals within a country. However, the technology aspects were modeled using a country-level indicator. This dimension could be explored in greater depth if more disaggregated information on indicators of technology use and development were obtained and modeled at different levels for each country. This is a subject for future research. The examination of disaggregated indicators of technology use and development would facilitate the identification of the specific factors that enable a country to be more innovative.

Furthermore, it is essential to acknowledge that the model is contingent upon a specific set of variables. Consequently, future research could investigate the potential incorporation of additional factors, such as the regulatory environment or business culture.
